# Dominant twin peaks: a novel conjecture for the pathophysiologic basis of tremor frequency and fluctuation time in Parkinson’s disease

**DOI:** 10.3389/fnins.2025.1398834

**Published:** 2025-06-18

**Authors:** Furrukh Khan, David Novikov, Brian Dalm, Jessie Xiaoxi, Oliver Flouty, Evan Thomas

**Affiliations:** ^1^Department of Electrical and Computer Engineering, The Ohio State University, Columbus, OH, United States; ^2^Department of Computer Science and Engineering, The Ohio State University, Columbus, OH, United States; ^3^Department of Neurosurgery, The Ohio State University, Columbus, OH, United States; ^4^Department of Neurosurgery and Brain Repair, University of South Florida, Tampa, FL, United States; ^5^Department of Radiation Oncology, The Ohio State University, Columbus, OH, United States

**Keywords:** Parkinson’s disease, tremor frequency, subthalamic nucleus, local field potentials, deep brain stimulation, functional neurosurgery border

## Abstract

**Background:**

With the commercial availability of deep brain stimulation neurostimulators and sensing leads capable of recording deep brain Local Field Potentials, researchers now commonly study the spectral characteristics of Local Field Potentials recorded from the subthalamic nucleus of patients with Parkinson’s disease. Correlating subthalamic synchronized oscillatory activity with motor impairment in Parkinson’s disease patients has recently gained attention in the literature.

**Objective:**

Based on the deep brain recordings of a Parkinson’s disease patient our objective is to (i) Use actual measurements of the patient’s tremor to support a hypothesis that connects the features of the Local Field Potential’s beta-band spectrum (13–31 Hz), with the lower frequency (4–8 Hz) features of the patient’s tremor, such as tremor frequency and tremor fluctuation time and (ii) Justify the hypothesis through theoretical reasoning based on communication theory in Electrical Engineering.

**Methods:**

Tremor characteristics (i.e., tremor frequency and tremor fluctuation time) derived from limb coordinate time-series were obtained from a video of the patient by using Google’s MediaPipe Artificial Intelligence Framework. Spectra of the deep brain recordings and measured tremor time-series were analyzed using the Fast Fourier Transform. Burst trains in the deep brain signals and tremor bursts in the measured tremor signal were investigated by using Continuous Wave Transform scalograms.

**Results:**

Support for the hypothesis is provided by a close agreement between the measured results of the tremor (from a patient’s video) and the predictions of the hypothesis based on the Local Filed Potential deep brain spectrum. We show that the defining features in the scalogram obtained from the deep brain signal are directly related to the features in the scalogram of the measured tremor. We provide a theoretical justification of the hypothesis by relating features of the deep brain beta-bursts, seen in the Local Field Potential scalogram, to a pair of beta-band dominant peaks found in the spectrum of the deep brain signal by leveraging the phenomena of “beating” (amplitude modulation) from communications theory.

**Conclusion:**

We conclude that tremor properties of a Parkinson’s disease patient, like tremor frequency and tremor fluctuation duration, can be obtained from the patient’s subthalamic nucleus beta-band spectrum.

## Introduction

With the commercial availability of Deep Brain Stimulation (DBS) neurostimulators and sensing leads capable of recording deep brain Local Field Potentials (LFPs), researchers now commonly study the spectral characteristics of LFPs recorded from the subthalamic nucleus (STN) of patients with Parkinson’s disease (PD) (Feldmann et al., 2022; Koeglsperger et al., 2021; Plate et al., 2021; Vissani et al., 2020; Neumann et al., 2016; Neumann et al., 2017; Telkes et al., 2018; Beudel et al., 2015; Tinkhauser et al., 2017a,b; Tinkhauser et al., 2018; Torrecillos et al., 2018). Exaggerated basal ganglia activity in the beta band is commonly found in patients with PD.

Recently, the peaks in the beta band power spectrum have gained attention in connection with using the peaks as biological feedback signals for closed-loop DBS in patients suffering from PD (Feldmann et al., 2022; Koeglsperger et al., 2021; Plate et al., 2021; Vissani et al., 2020). Extensive work has also been published to correlate subthalamic synchronized oscillatory activity with motor impairment in patients with PD (Neumann et al., 2016; Neumann et al., 2017; Telkes et al., 2018; Beudel et al., 2015). Bursts of LFP power in the beta band, known as beta bursts, have also been observed and studied recently (Tinkhauser et al., 2017a,b; Tinkhauser et al., 2018; Torrecillos et al., 2018).

Not all PD patients are tremor dominant (TD). In this paper, we focus only on the tremor dominant (TD) subtype of PD and the related beta burst trains. Specifically, we focus on resting tremor in PD patients. In Parkinson’s disease (PD), resting tremor refers to an involuntary shaking that occurs when the muscles are relaxed. This tremor most commonly affects the hands, but it can also involve other parts of the body, such as the arms, legs, face, or jaw. Typically, the tremor has a frequency between 4 and 8 Hz (Bain, 2002).

This paper presents a hypothesis based on the STN LFP recordings of a PD patient. To lay the grounds for the rest of this paper we first outline the central problem we are addressing. If we measure the frequency spectrum of the limb motions of a PD patient, for example by evaluating the video of the patient or using motion capture cameras, we find a strong peak in the measured spectrum. This peak is centered around the tremor frequency of the patient, *f*_*tremor_measured*_; the finite width of this peak, Δ*f*_*tremor_measured*_, is related to the time duration of the tremor fluctuations, *T*_*tremor_fluctation*_, i.e., the time interval over which the tremor’s intensity fluctuates, i.e., repeatedly waxes and wanes over time. Since the statistical population of PD patients has an average tremor frequency in the range 4 – 8 Hz (Bain, 2002) *f*_*tremor_measured*_ lies in this interval. In contrast, if we inspect the LFP spectrum, we observe that the beta band frequencies, 13 to 31 Hz, are much larger than *f*_*tremor_measured*_. Furthermore, the widths of the various peaks in the beta band seem to have no obvious connection to Δ*f*_*tremor_measured*_. There seems to be no clear relationship between the features of the LFP spectrum in the beta band and the measured tremor spectrum (*f*_*tremor_measured*_,Δ*f*_*tremor_measured*_). This paper proposes a novel hypothesis that makes a direct connection between the features of the (higher frequency) LFP beta band spectrum and (lower frequency) measured tremor spectrum features, *f*_*tremor_measured*_,Δ*f*_*tremor_measured*_, and *T*_*tremor_fluctation*_. To support our hypothesis, we independently measure the patient’s limb coordinates time-series obtained from a video of the patient. Google’s MediaPipe^1^ framework is used to obtain the limb coordinates corresponding to the patient’s left and right wrists, knees, and heels. The hypothesis will be presented formally after some background material has been covered.

In addition, the paper also presents a novel theoretical foundation on which the hypothesis is based, by noting that the LFP spectrum of our PD patient when appropriately smoothed, shows two dominant frequency peaks close to each other in the beta band. For the first time in the literature, we explicitly link the beta burst trains found in the STN local field potentials of a PD patient to a phenomenon known as “beating” (or amplitude-modulation) in the field of communications. In amplitude modulation two frequencies close to each other, when superimposed, give rise to a power burst train which has a frequency equal to the difference of the two superimposed frequencies. This resulting burst train has a lower frequency than the original two frequencies. We show that the lower-frequency burst trains seen in the time-frequency visualization of the patient’s LFP data arise from the beating of the two higher-frequency dominant peaks observed in the beta band.

Finally, we run a pair of large numerical simulations to investigate the likelihood of observing dominant twin peaks due to random chance only and conclude that, based on the results of these simulations, the dominant twin peak structure observed in our LFP data is unlikely to arise solely due to random noise.

## Materials

The LFP time series were obtained from a 71-year-old female patient suffering from PD. Medtronic’s SenSight™ directional (1-3-3-1) electrode leads,^2^ Model B33005™, were implanted into the dorsolateral aspect of the STN on both the left and right hemispheres. A Medtronic Model B33005 Percept™ PC neurostimulator with BrainSense™ technology was implanted on March 2023 to deliver DBS. The neurostimulator (in conjunction with directional leads) is capable of recording intracranial LFPs through one or two leads implanted in the brain. This implanted neurotransmitter was used with stimulation turned off to make the LFP signal recordings on March 2023.

### LFP recordings

**FIGURE 1:**
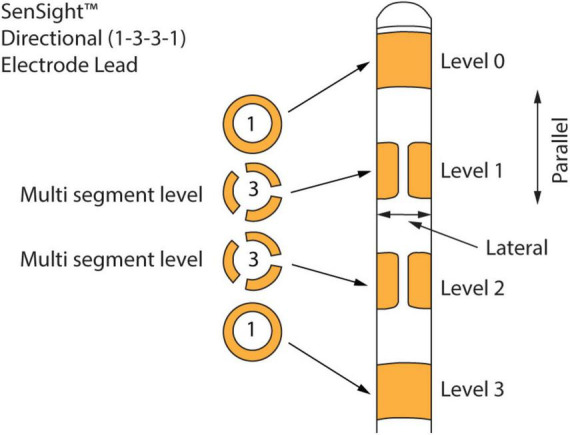
Medtronic 1-3-3-1 directional leads. There are four levels of electrodes along the length of the lead. The top and bottom electrodes are continuous conducting full rings, while the middle level electrodes are split into three isolated conducting segments. These leads allow stimulation and recording not only between levels (*parallel* to the leads) but also between segments of a multi-segment level (*lateral* direction to the leads).

The 1-3-3-1 leads, shown in Figure 1, are known as directional leads (see text footnote 2). Four levels (0, 1, 2, and 3) of electrodes are arranged along the length of a lead. All the available measurement directions are grouped into channels. There are 15 channels per lead, for a total of 30 channels if leads are implanted in both hemispheres (as is the case for the recordings in this paper). As explained in Figure 1, these leads allow stimulation and recording between levels (parallel to the leads) and between segments of a multi-segment level (*lateral* direction to the leads). The recordings were made with stimulation turned off. We download the LFP time series as a JSON file from the tablet that is used to communicate with the neurostimulator. The time series for each of the 30 channels consists of 21-s LFP recordings sampled at 250 Hz. Medtronic also applies two 100 Hz low-pass and two 1 Hz high-pass filters to the signal. We use these LFP signals for all the analyses in this paper.

### Beating of two higher frequencies to produce a lower frequency power burst

Here we illustrate the phenomena of beating or amplitude modulation by using simple mathematical reasoning. Consider the superposition of two sinusoids with frequencies *f_1_* and *f_2_* (which lie close to each other) by using the identity,


(1) $$\sin\,\left(2\,\pi \,f_{1}\,t\right)+\sin\,\left(2\,\pi \,f_{2}\,t\right)=$$

$$2\,s\,i\,n\,\left(2\,\pi \,\frac{f_{1}+f_{2}}{2}\,t\right)\,\cos\,\left(2\,\pi \,\frac{f_{1}-f_{2}}{2}\,t\right),$$

which shows that the superposition of two waves with similar frequencies *f_1_* and *f_2_* produces a carrier wave that oscillates with the frequency,


(2) $$\,f_{c\,a\,r\,r\,i\,e\,r}=\frac{f_{1}+f_{2}}{2},$$

and whose amplitude is modulated with a lower frequency wave with frequency,


(3) $$\,f_{m\,o\,d}=\frac{f_{1}-f_{2}}{2}.$$

The beat frequency is defined as the frequency of modulation of the power in the signal (square of the magnitude of the signal),


(4) $$\,f_{b\,e\,a\,t}=2\,f_{m\,o\,d}=f_{1}-f_{2}.$$

We note that the beat frequency *f*_*beat*_ is much lower than the frequencies *f_1_* and *f_2_*, particularly when these frequencies are close to each other.

### Tools used for signal processing and analysis

In this paper, Python v3.2.10 and its scientific packages are used for all signal processing and analysis of the signals. Fast Fourier Transforms (FFT) algorithm (McClellan et al., 2016) evaluated by using the scipy.fft.rfft function, is used to calculate the frequency spectra of the LFP signals as well as the limb tremor spectra obtained from the video of the patient. Frequency-bin sizes of 0.047 Hz for LFPs, and 0.05 Hz for the video recordings are employed. The square of the magnitude spectrum from the FFT only shows power at a particular frequency without any information about when a frequency event is happening in time. To get that information we also need the phase spectrum of the FFT. However, it is not possible to visualize time events by just looking at the magnitude and phase FFT spectra together. To visualize beta power bursts in detail we need to show the signal’s power both in the frequency and the time domains simultaneously while also obeying the time-frequency uncertainty principle (Addison, 2020; Mallat, 2008). We use the Continuous Wavelet Transform (CWT) scalogram of the LFPs time series for this visualization of the LFP signal and also the measured tremor time series. To concentrate on the beta band, the LFP signal is passed through a 13–31 Hz 5th-order discrete Butterworth band-pass filter. Butterworth filters are also known as *maximally flat magnitude filters* since they are designed to give a frequency response as flat as possible in the passband. Python’s function named cw with gaus8 wavelet from the pywt package was used to obtain the scalograms. We use 8 and 50 Hz for the upper and lower frequencies of the LFP scalograms; for the measured limb tremor scalograms, we use 1 and 10 Hz.

### Automatic (programmatic) detection of the dominant twin peaks in the LFP spectrum

To make the detection of the dominant twin peak in the LFP spectrum objective, we use software to detect the peaks in the spectrum automatically with a desired prominence (quality) from all the 30 channels. We use the find_peaks function of Python’s scipy.signal package with the prominence parameter set to 0.04. Before detecting the peaks programmatically, the FFT of the signal needs to be smoothed appropriately to reveal the dominant peaks. Note that we are smoothing the FFT spectrum in the frequency domain to specifically observe and detect the peaks in the FFT. A low pass filter that is designed to give a flat passband when used on a time series, such as the Butterworth filter, is not appropriate for this purpose since it potentially destroys the spectrum’s tenacity and smears the peaks, making it difficult to use software tools to detect the peaks. Instead, we use the Savitzky-Golay filter popularized by Whittaker and Robinson (1924) and Guest (2012) for smoothing the spectrum. This filter, popular in the signal processing community, works by fitting subsets (windows) of adjacent data points with low-degree (order) polynomials by using the least square fit method^3^ (Riordon et al., 2000). A window size of 51 and polynomial order of 3 is used in this paper. When we say that we have detected a twin peak in the spectrum of a channel, it is implied that our software has detected twin peaks with a permanence equal to or greater than 0.04.

### Measuring limb tremor frequencies from a video of the patient

**FIGURE 2:**
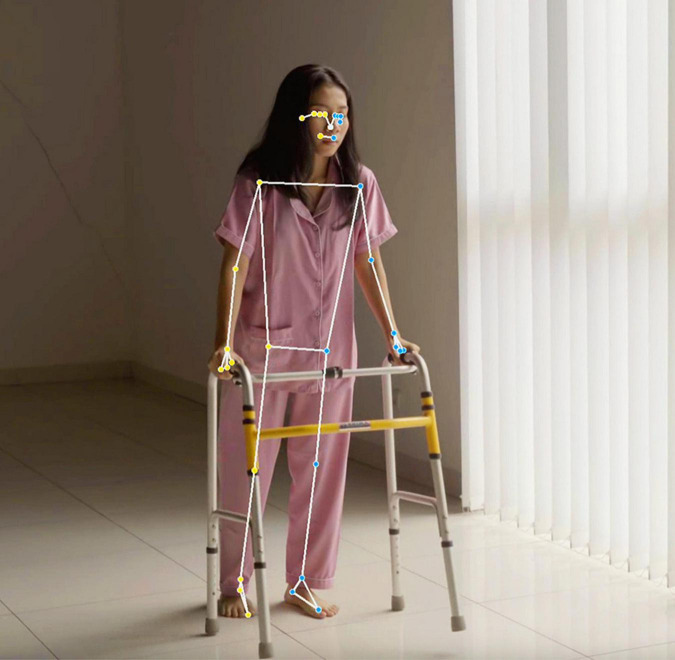
Key location points on a human body can be identified by Google’s MediaPipe’s “Pose Landmark Detection.” We use the right and left wrists, knees, and heels as key locations to determine the PD patient’s corresponding x and y coordinates from the patient’s video. The figure was generated from a stock video for illustrative purposes and is not from the PD patient’s video. The illustration in the figure is generated by Google’s MediaPipe from a video licensed from Adobe Stock.

Google’s MediaPipe (see text footnote 1) is a framework based on AI for developers to build multimodal (e.g., video or audio) machine learning pipelines. Its primary use case is to provide machine learning models that can run live on smartphones or other edge devices. It can be used to rapidly analyze videos of patients taken by mobile phones or tablets. In this paper, we make use of MediaPipe’s “Pose Landmark Detection” (see text footnote 1). It uses its internal AI models to automatically identify key location points on a human body in a video or an image. These points are located at major connections to limbs and torso, such as wrists, heels, and knees. The possible location points are illustrated in Figure 2 on a human body (see text footnote 1). The framework also automatically evaluates a time series of the x and y coordinates of the key location points from a video. This information is sufficient to measure the patient’s tremor frequency, *f*_*tremor_measured*_; either the x or y coordinate time-series can be used, and both give the same frequency characteristics. Since we are not interested in measuring the amplitude of the tremor in this paper, we do not require a time series for the z coordinates to account for the total motion trajectory of the limb. The limb tremor measurements are all obtained from a patient’s video made on December 15, 2022. A Sony HDR-CX440 Handycam video camera mounted on a tripod is used. The patient is seated on a sturdy chair with no other sources of vibration. The framework is used on this 854 by 480-pixel video, sampled at 30 fps (frames per second) to determine the x and y coordinates of the right and left wrists, knees, and heels of the PD patient.

## Hypothesis and results

The LFP signal measured by channel 17 is used to illustrate our results in most of the discussion in this paper. This channel is associated with a *lateral* measurement of the LFP between level-2 segments, a and c, of the left lead. This channel is denoted as “(17) 2a-2c left.” We chose this channel to illustrate our points because it shows the strongest beta band energy compared with the other channels. This will be discussed in detail in the section *Energy Distribution of LFPs from Different Channels* below where we calculate the energy in the alpha, beta, and gamma bands for all the 30 channels from the LFP spectrum. We point out that all the 30 channels, and not just channel number 17, were used to calculate the relevant statistical parameters which enabled us to reach the conclusions in this paper.

### Local field potential spectrum and scalogram

**FIGURE 3:**
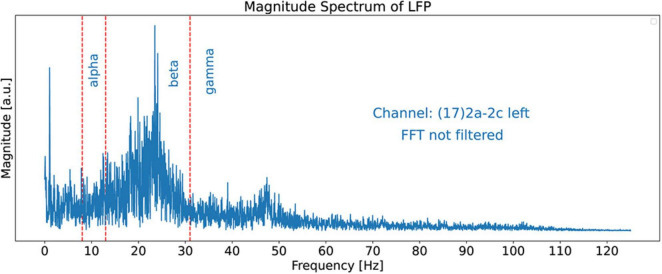
FFT Magnitude spectrum of the signal obtained from channel 17. This is a “regular” plot and not a stem (or lollipop) plot. The red dashed lines show the bounds of the alpha, beta, and gamma spectral bands at 8, 13, and 31 Hz, respectively. The figure does not exhibit dominant peaks clearly because no smoothing filter has been applied to the FFT signal in this figure.

In this paper, we use 21.15 s of channel 17’s LFP recorded signal with 5288 time-samples. The sampling rate is 250 Hz. Figure 3 shows the magnitude spectrum of this signal obtained by using the Fast Fourier Transforms (FFT) algorithm (McClellan et al., 2016). The bounds of the alpha, beta, and gamma spectral bands are shown by red dashed lines. For the boundaries of the spectral bands, we use 8 Hz, 13 Hz, and 31 Hz for the alpha, beta, and gamma bands, respectively. Note that it is hard to detect dominant peaks in the signal, especially by software tools, because of the lack of smoothness.

#### Tremor frequency’s relationship with the LPF scalogram

**FIGURE 4:**
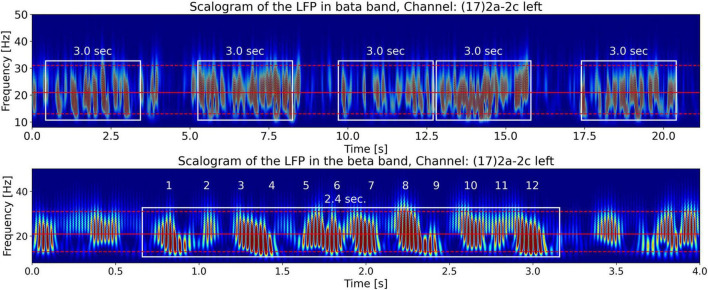
CWT scalograms (Addison, 2020) of beta-band LFP signal power from channel 17. The beta-band signal is obtained by passing the time signal through a 13–31 Hz, 5th-order discrete Butterworth band-pass filter. The top and bottom dashed red horizontal lines are the edges of the beta band. The middle solid red horizontal line is at the carrier frequency illustrated in Figure 9. The *top plot* contains 21.15 s of LFP activity. It shows that beta burst trains appear in burst groups with a burst group duration of around 3 s (enclosed in white rectangles of 3 s width). The *bottom plot* shows the first 4 s of LFP activity by zooming in on one burst group. The white rectangle encompassing 12 numbered semi-periodic bursts is 2.4 s wide indicating an approximate mean burst train frequency of ∼5 Hz.

Figure 4 shows the Continuous Wavelet Transform (CWT) scalogram (Addison, 2020; Mallat, 2008) of channel 17’s beta-band LFP signal power. We notice that the beta band signal power is composed of groups of bursts of LFP energy (enclosed in the white rectangles in the top plot), with each group composed of semi-periodic trains of bursts (enclosed in the white rectangle in the bottom plot). The top plot contains 21.15 s of LFP activity. It shows that the burst groups (enclosed in white rectangles) have an approximate duration of around 3 s. This burst group duration of the LFP data is one of the features we are interested in this paper and use *T*_*lfp_groups*_ to denote this time. Later in the paper, our hypothesis will connect this LFP feature, *T*_*lfp_groups*_, with the measured tremor fluctuation time *T*_*tremor_fluctation*_ from the video of the patient.

The bottom plot zooms-in on one semi-periodic burst train within a group by displaying the scalogram of the first 4 s of LFP activity. The white rectangle encompassing 12 numbered quasi-periodic bursts is 2.5 s wide, a quick division shows that these energy bursts have an approximate periodicity of 5 Hz. In the following sections we will explain the origin of the burst groups in the top figure and the burst trains in the bottom figure from the LFP spectrum. By observing the frequency axis of the scalogram we note that the frequencies making up the bursts (∼20 Hz) lie in the beta-band, however, as we have seen by counting the number of bursts along the time axis, the burst trains occur at a lower frequency of approximately 5 Hz. This is indicative of the phenomena known as beating or amplitude modulation in signal processing, explained in the *Materials* section above, when two superimposed high-frequency waves of similar frequencies beat against each other to produce lower-frequency modulated power bursts.

#### Dominant twin peaks in the filtered LPF spectrum

**FIGURE 5:**
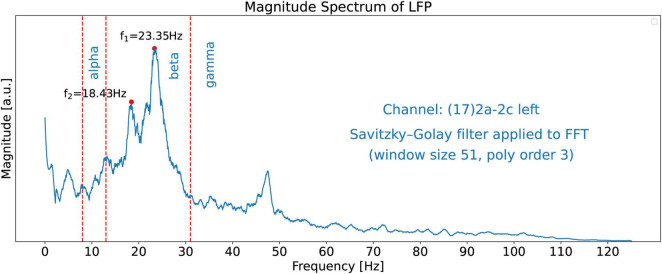
FFT magnitude spectrum of the signal obtained from channel 17. A Savitzky-Golay filter has been applied to the raw FFT shown in Figure 3 to unveil the salient features of the spectrum, such as the two dominant peaks at frequencies *f_1_* = 23.35 Hz and *f_2_* = 18.43 Hz.

The non-smooth nature of the FFT shown in Figure 3 makes it difficult to identify dominant peaks in the spectrum, particularly by software. To reveal the salient features of the spectrum we apply a Savitzky-Golay filter (see text footnote 3) (Whittaker and Robinson, 1924; Guest, 2012; Riordon et al., 2000) the FFT spectrum which then clearly reveals two dominant twin peaks in the beta band as shown in Figure 5. The dominant beta-band twin peaks in Figure 5 are automatically detected by our software to be *f_1_* = 23.35 Hz and *f_2_* = 18.43 Hz.

#### Idealized twin peaks model

**FIGURE 6:**
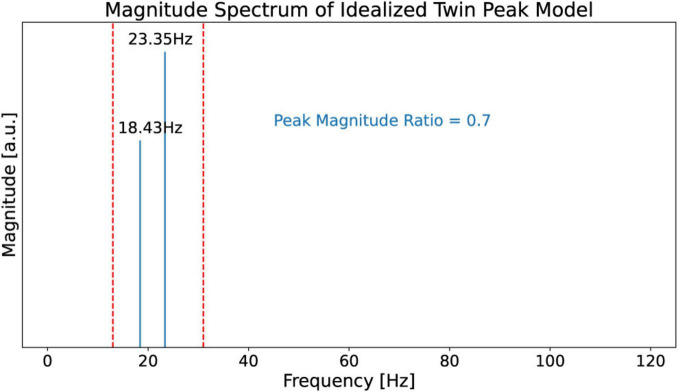
Magnitude spectrum of the idealized twin peak model. This model consists of only two frequencies *f_1_* = 23.35 Hz and *f_2_* = 18.43 Hz obtained from Figure 5. It also carries over the peak magnitude ratio of 0.7 from Figure 5.

**FIGURE 7:**
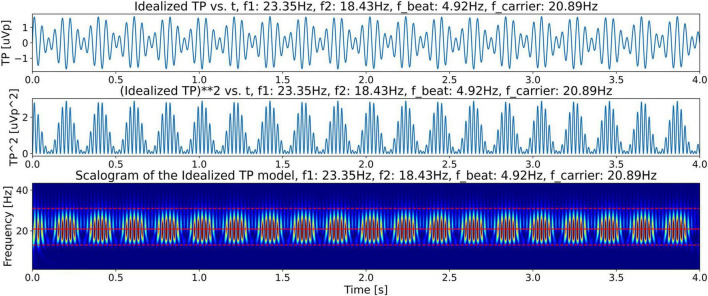
Illustration of beating in the idealized twin peak model. Top plot shows beating or amplitude modulation, illustrating how constructive and destructive superpositions of *f_1_* and *f_2_* signals produce beating phenomena. The middle plot shows the power in the signal (square of the magnitude). This plot shows bursts with frequency equal to the beat frequency, *f*_*beat*_. The bottom plot shows the CWT scalogram (Addison, 2020) of the signal power in the Idealized Twin Peak model showing the periodic burst train located in the beta band with a frequency equal to the beat frequency.

To support the claim that the beta burst trains in the LFP scalogram in Figure 4 are originating from the beating of the two dominant peak frequencies, we first construct an idealized model for illustrative purposes with contains only two frequencies which beat against each other. Specifically, we use this model to observe and recognize the signature of the beating of two frequencies in a CWT scalogram. This model, shown in Figure 6, consists of only two frequencies, *f_1_* = 23.35 Hz and *f_2_* = 18.43 Hz, obtained from the peaks in Figure 5. The corresponding time domain signal consists of a superposition of the two sinusoidal waves with frequencies *f_1_* and *f_2_*. This superposition that gives us the amplitude-modulated wave, Equation 1, is shown in the top plot of Figure 7, illustrating how constructive and destructive superpositions of the two sinusoidal signals produce the beating phenomena. The square of the magnitude of this signal which has a periodicity of the beat frequency, Equation 4, is shown in the middle plot of Figure 7. Finally, the bottom plot of Figure 7 shows the CWT scalogram (Addison, 2020) of the Idealized Twin Peak model which shows the characteristic signature of a burst train caused by the beating of two waves with frequencies *f_1_* and *f_2_*. The bursts in the idealized model occur periodically at the beat frequency of *f*_1_ − *f*_2_ = 4.92 Hz.

**FIGURE 8:**
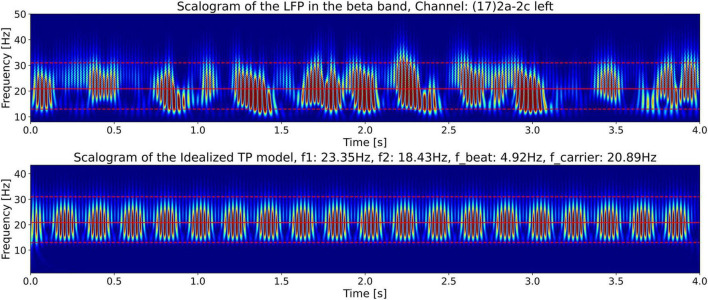
The top plot shows the CWT scalograms (Addison, 2020) of the LFP signal power from Figure 4 (bottom plot), and the bottom plot shows the scalogram of the Idealized Twin Peak Model from Figure 7, plotted together for ease of comparison.

**FIGURE 9:**
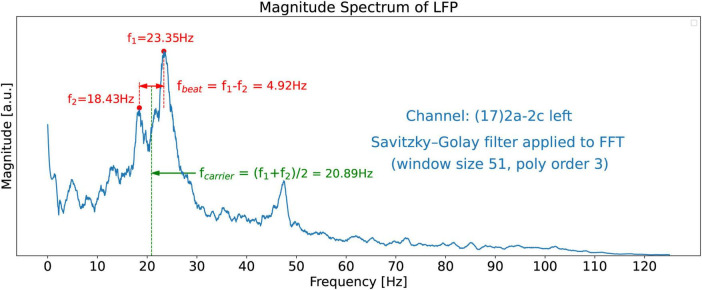
FFT Magnitude spectrum of the signal obtained from channel 17. Association of the two beta-band dominant peaks in the LFP Spectrum with two frequencies *f_1_* and *f_2_* which beat against each other to produce the beat frequency, *f_beat_*. The carrier frequency, *f_carrier_*, of the beats (burst trains), as well as *f_1_* and *f_2_* are in the beta band while *f_beat_* = 4.92 Hz is at a much lower frequency.

For a comparison of the CWT scalogram of the beta-band LFP and the Ideal Twin Peak Model, the corresponding scalograms of the two signals are drawn together in Figure 8. As pointed out earlier, to concentrate on the beta band, the LFP signal is passed through a 13–31 Hz 5th-order discrete Butterworth band-pass filter. Notice that the scalogram of the beta-band LFP signal (Figure 4) has the same signature of beta burst trains as exhibited in the scalogram of the Idealized Model which originated from the beating of two frequencies. Furthermore, the burst in the beta-band LFP scalogram also occurs periodically at the same beat frequency of *f*_1_ − *f*_2_ = 4.92 Hz. Figure 9 illustrates the association of the two beta-band dominant peaks in the LFP Spectrum. The two frequencies *f_1_* and *f_2_* beat against each other to produce the beat frequency, *f_beat_* = 4.92 Hz of the burst trains. The carrier frequency, *f_carrier_*, of the beats, as well as *f_1_* and *f_2_* are in the beta band while *f_beat_* is at a much lower frequency.

**FIGURE 10:**
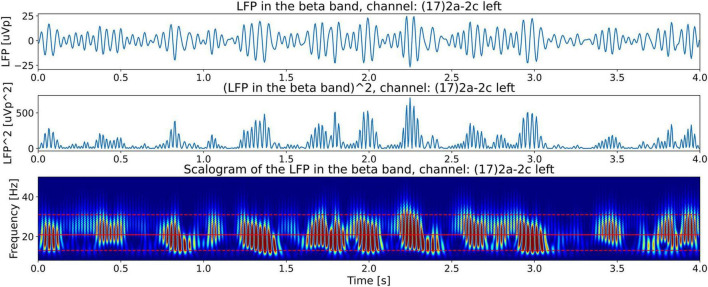
Illustration of beating phenomena in the beta band LFP obtained from channel 17. The top plot shows beating or amplitude modulation. The middle plot shows the power in the signal (square of the magnitude). This plot shows bursts with a mean frequency equal to the beat frequency, *f*_*beat*_. The bottom plot shows the CWT scalogram (Addison, 2020) of the beta band power in the LFP (same as bottom plot of Figure 4) showing the almost periodic burst trains located in the beta band with a frequency equal to the beat frequency, *f*_*beat*_.

Figure 10 further demonstrates the beating phenomena in the beta-band LFP signal. The top plot shows the beta-band signal vs. time, and the middle plot shows its power vs. time. The beating of the two frequencies *f_1_* and *f_2_* to produce semi-periodic beta bursts (because of constructive and destructive interference) is apparent in these plots. Finally, the bottom plot of Figure 10 shows the beta-band scalogram (same as in Figure 4). It is instructive to compare this figure with Figure 7, the equivalent figure for the Idealized Twin Peak model. The similarity between the two figures is apparent. We conclude that the wave train in the LFP scalogram not only has the signature of the beating of two frequencies but also the frequency of the power bursts equals *f*_1_− *f*_2_ where *f_1_* and *f_2_* are the dominant pair of peaks in the LFP spectrum.

### Statement of the hypothesis

Having laid the theoretical background, we now address the central problem mentioned at the beginning of this paper and connect the LFP spectrum to the spectrum of tremor by formally forming a hypothesis in three parts.

(a) We hypothesize that the frequency of the power burst trains, in the LFP scalogram (which we have shown to be *f*_1_ − *f*_2_, is equal to the tremor frequency *f*_*tremor_measured*_. In fact, the limb tremor of a patient is not composed of a single frequency, instead, the tremor spectrum consists of a peak located at *f*_*tremor_measured*_ with a non-zero width Δ*f*_*tremor_measured*_. Therefore, we calculate *f*_1_ − *f*_2_ over all the channels exhibiting dominant twin peaks to evaluate the mean (*f*_1_−*f*_2_)_*mean*_. The hypothesis associates this mean value with *f*_*tremor_measured*_,


(5a) $$\,f_{t\,r\,e\,m\,o\,r\,\_\,m\,e\,a\,s\,u\,r\,e\,d}={\left(f_{1}-f_{2}\right)}_{m\,e\,a\,n}$$

(b) The second part of the hypothesis links Δ*f*_*tremor_measured*_ to the LFP spectrum. We evaluate *f*_1_ − *f*_2_ over all the channels exhibiting dominant twin peaks and calculate the standard deviation (*f*_1_−*f*_2_)_*std dev*_. The hypothesis associates the standard deviation with Δ*f*_*tremor_measured*_,


(5b) $$\Delta \,f_{t\,r\,e\,m\,o\,r\,\_\,m\,e\,a\,s\,u\,r\,e\,d}={\left(f_{1}-f_{2}\right)}_{s\,t\,d\,d\,e\,v}$$

(c) We show later that the tremor fluctuation time, *T*_*tremor_fluctation*_, can be evaluated from Δ*f*_*tremor_measured*_. The third part of the hypothesis states that *T*_*tremor_fluctation*_is equal to the time duration, *T*_*lfp_groups*_, of groups of beta-band trains seen in the scalograms of the LFP (top plot, Figure 4),


(5c) $$\,T_{t\,r\,e\,m\,o\,r\,\_\,f\,l\,u\,c\,t\,a\,t\,i\,o\,n}=T_{l\,f\,p\,\_\,g\,r\,o\,u\,p\,s}$$

In summary, our hypothesis connects (predicts) three defining features of the measured tremor spectrum, *f*_*tremor_measured*_, Δ*f*_*tremor_measured*_, and *T*_*tremor_fluctation*_, to three independent features, (*f*_1_−*f*_2_)_*mean*_, (*f*_1_−*f*_2_)_*std dev*_ and *T*_*lfp_groups*_, seen in the LFP signals.

### Energy distribution in the LFP spectrum bands for different channels

**FIGURE 11:**
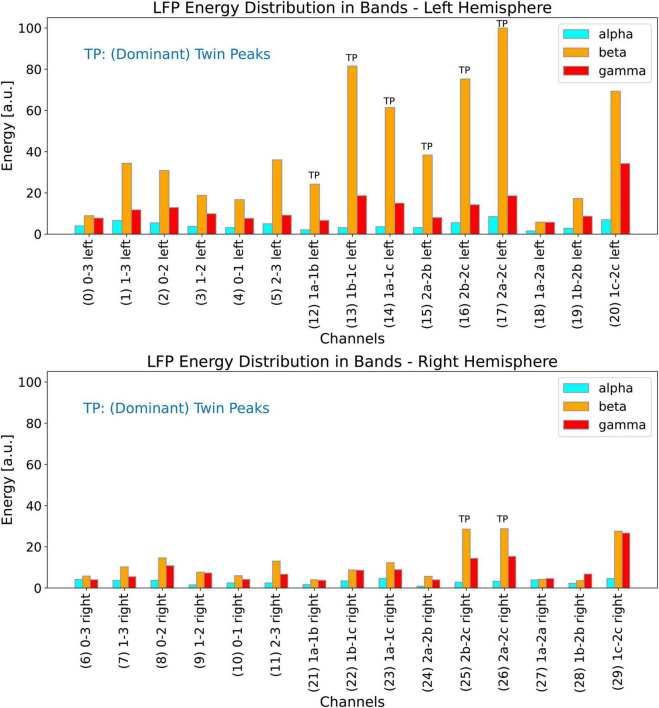
Distribution of energy of the LFP spectrum among the LFP alpha, beta, and gamma bands for the left (top plot) and right (bottom plot) channels. Both the histograms are drawn to the same scale so they can all be compared with each other. Channels marked with the letters TP exhibit dominant twin peaks which can be identified by our peak-finding algorithm.

In this section, we investigate the distribution of energy of the LFP spectrum among the alpha, beta, and gamma bands for all the 30 channels. We define the LFP energy in a band as the integrated power of the spectrum over the frequency range of that band. Figure 11 (top plot) shows the distribution of the energy for all the 15 channels of the left lead, and Figure 11 (bottom plot) shows the same results for the 15 channels of the right lead. The histograms are drawn to the same scale for all the 30 channels. For our patient, the LFP energy in the bands is much higher in the left channels than in the right channels.

#### Automatically picking channels with dominant twin peaks

We use the methods to detect the twin peaks in the LFP spectrum automatically as described in the materials section above. Figure 11 (top plot) shows all channels from the left hemisphere in which twin peaks were detected; these channels have been marked by the letters TP (Twin Peaks). It is interesting to note that these six channels comprise all the left *lateral* channels. Dominant Twin Peaks are weak or non-existent in measurements associated with all the other left channels, i.e., those that make left measurements *along the length* of the 1-3-3-1 lead. Since the LFP activity is lower in the right hemisphere, our automatic peak finding algorithm was able to identify only two channels in the right lead which exhibit clear Dominant Twin Peaks, marked by TP in Figure 11 (bottom plot). It should be noted that both these channels are also *lateral*. In summary, none of the left or right channels that make measurements *along the length* of the leads show Dominant Twin Peak activity that could be automatically detected by our software. We emphasize this point so that to replicate the results of this paper, other researchers may need to ensure that lateral channels for DBS recordings are also selected.

### Evaluating LFP statistical parameters

**FIGURE 12:**
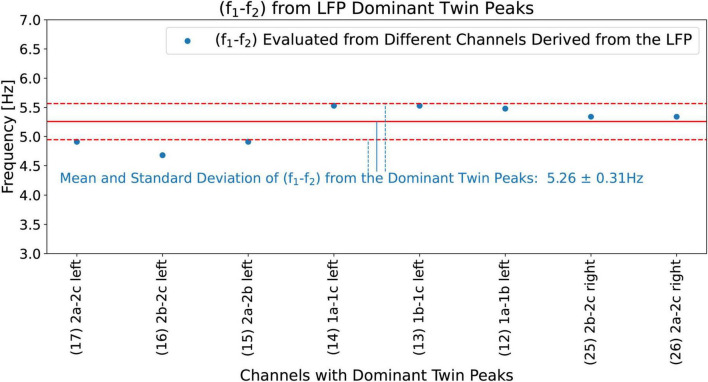
(*f*_1_−*f*_2_) calculated from all the eight channels that show Dominant Twin Peak activity (marked by TP in Figure 11). These give (*f*_1_−*f*_2_)_*mean*_ = 5.26 Hz (solid red line) and a standard deviation (*f*_1_−*f*_2_)_*std dev*_ = 0.31 Hz (dashed red lines showing +- standard deviation).

Now we evaluate the mean (*f*_1_−*f*_2_)_*mean*_, and the standard deviation (*f*_1_−*f*_2_)_*std dev*_ by using all the eight channels that show Dominant Twin Peak activity (marked by TP in Figure 11). The results are shown in Figure 12, with a mean (*f*_1_−*f*_2_)_*mean*_ of 5.26 Hz, and a standard deviation of deviation (*f*_1_−*f*_2_)_*std dev*_ of 0.31 Hz. Hence, based on Equations 5a, b, our hypothesis predicts a tremor frequency for the PD patient to be 5.26 ± 0.31 Hz. We note that this width, 0.31 Hz, has no obvious relationship with the width of the peaks in the beta band of the LFP spectrum.

### Measurements of the limb tremor characteristics from a video of the patient

**FIGURE 13:**
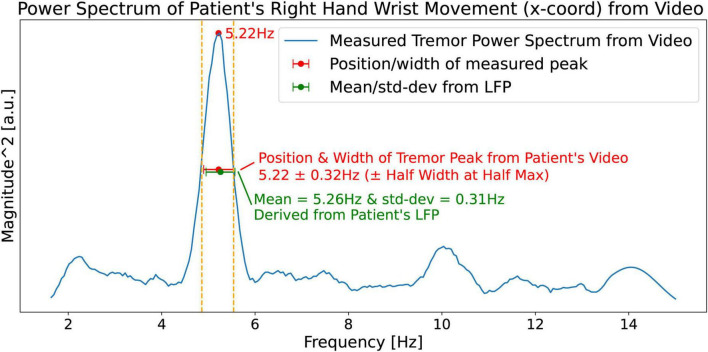
Measured (from the patient’s video) power spectrum (blue curve) of the patient’s right hand’s wrist movement showing a right wrist tremor peak located at *f*_*tremor_measured*_ = 5.22 Hz and a Half Width at Half Max (HWHM) of Δ*f*_*tremor_measured*_ = 0.32 Hz. The predictions based on our hypothesis (Figure 12) are shown in green.

Google’s MediaPipe (see text footnote 1) framework was used to obtain a time series consisting of 550 time-samples (18.33 s) of the x and y coordinates of the limbs from a video of the patient. Figure 13 shows the tremor power spectrum (square of magnitude FFT) obtained from the time series of the PD patient’s right hand’s wrist movement. The spectrum shows a right wrist tremor peak located at *f*_*tremor_measured*_ = 5.22 Hz, and a Half Width at Half Max (HWHM) width Δ*f*_*tremor_measured*_ = 0.32 Hz. Predictions made by our hypothesis, Equations 5a, b, are shown in green. Note that the measured quantities *f*_*tremor_measured*_ = 5.22 Hz, and Δ*f*_*tremor_measured*_ = 0.32 Hz, show a close match with the predictions of our hypothesis 5.26 ± 0.31 Hz obtained from the LFP (Figure 12).

#### Measured tremor frequencies from the wrists, knees, and heels

**FIGURE 14:**
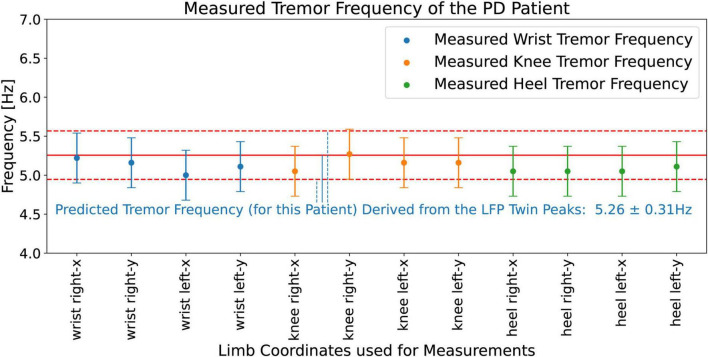
Measured (from the video) tremor frequencies, *f*_*tremor_measured*_, from the x and y coordinates corresponding to the right and left wrists, knees, and heels of the PD patient. The vertical error bars on the data points show the widths of the measured tremor peaks, Δ*f*_*tremor_measured*_. Solid red line shows the (hypothesis) predicted mean, 5.26 Hz, while the pair of horizontal red dashed lines show the upper and lower bounds, ± 0.31 Hz, of the prediction.

Figure 14 shows the measured (from the video) tremor frequencies, *f*_*tremor_measured*_, from the x and y coordinates of the right and left wrists, knees, and heels of the PD patient, and compares these measurements with the hypothesis predictions, 5.26 ± 0.31 Hz (Figure 12), obtained from the LFP Dominant Twin Peaks. The vertical error bars show the widths of the measured tremor peaks, Δ*f*_*tremor_measured*_. The agreement between the two is excellent; all eight measurements lie within the margins of the hypothesis predictions based on the LFP spectrum. These results lend strong credence to our hypothesis predictions, Equations 5a, b, by demonstrating that the tremor frequencies based on the beta-band Dominant Twin Peaks not only match, *f*_*tremor_measured*_, the location of the measured tremor frequencies, but also the measured widths, Δ*f*_*tremor_measured*_, of the tremor frequency peaks.

#### Determining the tremor fluctuation time from the video

We now focus on the third part of our hypothesis, Equation 5c, and first determine Δ*f*_*tremor_measured*_ from the video data and calculate the tremor fluctuation time, *T*_*tremor_fluctation*_ from it. Based on Fourier Transform theory, a non-zero spectral width implies that the signal has a corresponding finite duration σ_*t*_ (Mallat, 2008),


(6) $$\sigma_{f}\sigma_{t}∼\;\frac{1}{4\,\pi },$$

where, σ_*f*_ is the sigma (standard deviation) of the frequency peak, and σ_*t*_ is the sigma (standard deviation) of the time peak. We define Δ*f*_*tremor_measured*_ as the Half Width at Half Max (HWHM) of the tremor peak. For a Gaussian, HWHM = Δ*f*_*tremor_measured*_ = 1.2σ_*f*_. We use a 5-sigma half width for the duration Δ*t*_*tremor_measured*_, which gives,


(7) $$\Delta \,t_{t\,r\,e\,m\,o\,r\,\_\,m\,e\,a\,s\,u\,r\,e\,d}∼\frac{3}{\pi \,\Delta \,f_{t\,r\,e\,m\,o\,r\,\_\,m\,e\,a\,s\,u\,r\,e\,d}}.$$

A finite Δ*t*_*tremor_measured*_implies that the tremor intensity is fluctuating (waxing and waning) over this interval, i.e., it gives us the measured tremor fluctuation time (from the video),


(8) $$T_{t\,r\,e\,m\,o\,r\,\_\,f\,l\,u\,c\,t\,a\,t\,i\,o\,n}=\Delta \,t_{t\,r\,e\,m\,o\,r\,\_\,m\,e\,a\,s\,u\,r\,e\,d}$$

Using Δ*f*_*tremor_measured*_ = 0.32 Hz from Figure 13, Equations 7, 8 give *T*_*tremor_fluctation*_ = 3.0 sec.

#### Measured scalogram from the patient’s video

**FIGURE 15:**
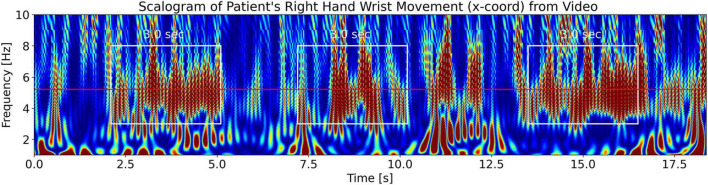
Measured CWT scalogram (Addison, 2020) of the patient’s right-hand wrist tremor obtained from the video of the patient. The middle solid red horizontal line is the measured tremor frequency, *f*_*tremor_measured*_ = 5.22 Hz, from Figure 13. The scalogram shows that the tremor (with frequency 5.22 Hz) has a fluctuating intensity in time of approximately *T*_*tremor_fluctation*_ = 3.0 s durations (enclosed by the white rectangles of 3.0-s width).

To visualize tremor fluctuations in the measured tremor signal from the video of the patient, we evaluate the signal’s CWT scalogram (Addison, 2020). Figure 15 shows the scalogram for the PD patient’s right wrist movement. We see the patient’s tremor burst trains (enclosed in white rectangles) which are located at a frequency of 5.22 Hz (the horizontal red line at 5.22 Hz is the measured tremor frequency from Figure 13). We can also get a back-of-the-envelope estimate of the tremor frequency by counting the number of maxima (dark red features) in a white rectangle to be approximately 30 features per 3.0 s. Dividing this by 3.0 s and further dividing by 2 (since the scalogram is a power spectrum) gives ∼5 Hz, a close approximation to the measured tremor frequency. We note that the scalogram shows *T*_*tremor_fluctation*_=3.0 sec. fluctuations in the intensity of the tremor (enclosed in white rectangles). Scalograms from the other limbs for x and y coordinates also show the same behavior. We compare this to the LFP signal power scalogram in Figure 4 (top plot), which shows that the LFP burst groups also have durations, *T*_*lfp_groups*_, of approximately 3.0 s, lending support for the third part of our hypothesis, Equation 5c.

### Likelihood of observing dominant twin peaks due to random chance

We restrict ourselves to the beta-band and ask this question: Can the spectral twin peaks observed in this paper be caused by the presence of random noise in the spectrum? In other words, if we start with a magnitude spectrum which contains one *dominant peak* at frequency *f_1_*, with *prominence* ≥ *0.04*, then what is the statistical likelihood that noise in the spectrum alone can cause another *dominant peak* with frequency *f_2_* to appear with *prominence* ≥ 0.04, which also satisfies the condition that |f_1_−f_2_| *lies within the range:*


(9) $$\left[f_{t\,r\,e\,m\,o\,r-m\,e\,a\,s\,u\,r\,e\,d}-2\,\sigma_{f},f_{t\,r\,e\,m\,o\,r-m\,e\,a\,s\,u\,r\,e\,d}+2\,\sigma_{f}\right]=\left[4.68,5.76\right]\,H\,z\,?$$

Where σ_*f*_ (standard deviation of the measured peak) was evaluated by using the relationship HWHM = Δ*f*_*tremor_measured*_ = 1.2σ_*f*_ mentioned earlier in the last section, and Half Width at Half Max (HWHM) was read off from the measured magnitude spectrum (Figure 13).

We address this question in two different ways by performing a pair of simulations comprising a large number of surrogate trials. In each of these trials, we simulate the noise in the spectrum to have a Root Mean Square (RMS) value consistent with noise exhibited in our LFP spectra; specifically, we choose channel 5 because it exhibits a dominant single peak with prominence ≥ 0.04. We calculate the LFP noise in the beta band of channel 5 by subtracting the raw noisy LFP spectrum obtained from channel 5 from the smoothed (Savitzky-Golay filter) LFP spectrum. The calculated RMS = 0.08 value of this subtracted signal (in the beta-band) is then used to generate uniform noise for all the trials in our simulations. As explained below, for *simulatin-1* we superimpose noise onto an A/*f*^α^ spectrum (known as 1/*f* decay) fitted to channel 5, while for *simulation-2*, we add noise directly to the smoothed spectrum of channel 5 with the pre-existing dominant peak.

*Simulation-1:* We note that neural LFP signals commonly exhibit a 1/*f* decay in their power spectrum (Bédard and Destexhe, 2009), which follows a straight line on a log-log plot of the power spectrum vs. frequency. On a linear plot, this leads to a magnitude spectrum with a *A*/*f*^α^ (Smith, 2011, Chapter 1.7.3) behavior, where *A* and α are obtained by fitting to LFP data. We determine these parameters by fitting a straight line to the log(power spectrum) vs. log(frequency) plot of channel 5’s spectrum. This fit gives us a slope *m* = −0.85 and an intercept *b* = −1.21. From these, we obtain the parameters for the magnitude spectrum, *A*=*e^b/2^* = 0.55 and α = −*m*/2=0.42. Using a Python program, we generate 100,000 surrogate trials. A magnitude spectrum is constructed in each trial by generating uniform noise consistent with the desired RMS value and superimposing it onto the magnitude spectrum *A*/*f*^α^. To detect peaks in each of the trials, we apply the Savitzky-Golay filter (as is done throughout this paper) and use our peak detection algorithm in the beta band with the prominence parameter set to 0.04. To detect twin peaks, we use the criterion |f_1_−f_2_|≤[4.68, 5.76]*Hz*. Out of a total of 100,000 surrogate trials, we extract the following statistics (CI stands for 99% Clopper–Pearson Confidence Intervals (CI) based on the 100,000-trial simulation):

512 trails are detected with twin peaks among 56,189 trails with at least one peak with prominence ≥ 0.04.

Conditional probability of observing a twin peak given that at least one prominent peak exists in the magnitude spectrum: 0.009112 (0.91%)

99% CI: [0.00811, 0.01019]

512 trials are detected with twin peaks among a total of 100,000 trials

Unconditional probability of detecting a twin peak: 0.00512 (0.51%)

99% CI: [0.00455, 0.0057]

The Clopper-Pearson method uses the Cumulative Distribution Function (CDF) of the binomial distribution to get precise bounds and is especially useful when dealing with a small number of successes. We use consistent units for the spectra throughout this analysis.

*Simulation-2:* In this simulation (instead of using 1/*f* decay), we start with the smoothed channel 5’s spectrum (Savitzky-Golay filter), which already has a dominant peak at frequency *f*_1_=22.83*Hz*, and generate 40,000 surrogate trials. Similar to *simulation_1*, we add noise to this spectrum in each trial. We note that the prominence criterion of a peak is not reliant on the height of the peak; instead, it only considers how well the peak stands out compared with its immediate surroundings. Since the existing peak *f_1_* in the spectrum is quite robust (dominant) we want to ensure that the random noise peak *f_2_* we detect in the trials not only has prominence ≥ 0.04 but is also robust (high enough compared with *f_1_*) to exhibit the beating phenomena on which our hypothesis is based, i.e., it (in conjunction with *f_1_*) can produce a detectable and observable tremor frequency of |f_1_−f_2_| as seen in the video of the patient. Therefore, on top of the conditions for detecting peaks and twin peaks used in *simulation-1*, we add the following condition on the random peak’s height: 0.6*f*_1−*height*_ < *f*_2−*height*_ < 1.4*f*_1−*height*_. This condition is satisfied by all the LFP twin peaks detected by our algorithm in this paper. Out of a total of 40,000 surrogate trials, we extract the following statistics:

743 successful trials out of a total of 40,000 trials.

Probability of detecting |f_1_−f_2_|≤[4.68, 5.76]*Hz*, i.e., a twin peak: 0.0185 (1.8%)

99% CI: [0.01688, 0.02038]

Based on the results of these simulations, we conclude that the dominant twin peak structure observed in our LFP data is unlikely to arise by chance due to random noise only.

## Discussion

### Conclusion

In this paper, we provide strong support for our hypothesis comprising Equations 5a–c. Specifically, for the first time in the literature, we connect features of the beta band spectrum with the defining features associated with the tremor dynamics measured from the patient’s video, such as tremor frequency and tremor fluctuation time. This connection is between two spectra that lie in different frequency bands; beta-band STN LFPs lie in the 13-31 Hz range while the physical tremors of PD patients lie in the 4–8 Hz (Bain, 2002) range. Furthermore, beyond the hypothesis itself, we also use communication theory to theoretically explain this connection between the two spectra by asserting that the tremor frequency oscillations are due to the beating of two dominant frequencies in the beta band of the STN LFPs.

To summarize, the paper concludes that properties of a PD patient’s tremor such as frequency and tremor duration can be obtained from the patient’s subthalamic nucleus beta-band spectrum. Furthermore, we conclude that such a dominant twin peak structure is unlikely to arise by chance only.

### Relevance, limitations, and future objectives

The work in this paper sheds light on new possibilities for interpreting and utilizing DBS recordings of PD patients. Furthermore, the identification of beta-band dominant twin peaks, specifically in the lateral channels of directional leads, may suggest new directions in electrode design and application. This specificity may pave the way for enhanced precision in signal reading, possibly leading to more effective treatment delivery. The dominant Twin Peaks may also have implications for research in closed-loop DBS (Feldmann et al., 2022; Koeglsperger et al., 2021; Plate et al., 2021; Vissani et al., 2020) for PD patients. The utilization of Google’s MediaPipe AI (see text footnote 1) framework introduces a simple approach to measuring tremor characteristics without the need for complex multiple-camera motion capture systems.

While the match between the predictions of our hypothesis and actual tremor measurements demonstrated in this paper is remarkable, we acknowledge the limitations of a single-patient study, since it cannot address questions, to be addressed in future research, like: (i) Are dominant Twin Peaks detected by using the methods presented in this paper found in the STN DBS recordings of all PD patients exhibiting tremors? (ii) Is our hypothesis comprising Equations 5a–c valid for all PD patients with tremors? (iii) Can these results be extended to Essential Tremor (ET) patients? Therefore, after receiving IRB (Institutional Review Board) approval, we are preparing to apply our research methodology to a larger cohort of patients.

## Data Availability

The raw data supporting the conclusions of this article will be made available by the authors, without undue reservation.

## Footnotes

1 Google MediaPipe Pose Landmark Detection Guide: https://developers.google.com/mediapipe/solutions/vision/pose_landmarker

2 Medtronic SenSight™ directional leads: https://www.medtronic.com/us-en/healthcare-professionals/products/neurological/deep-brain-stimulation-systems/sensight-lead.html

3 Savitzky-Golay filter, Wikipedia: https://en.wikipedia.org/wiki/Savitzky%E2%80%93Golay_filter#cite_note-analytchemsem-7
